# Research on the health of and interventions for family caregivers of people with dementia: a bibliometric analysis of research output during 1988–2018

**DOI:** 10.1186/s12877-020-1421-7

**Published:** 2020-01-21

**Authors:** Hui Shi, Chen Mao, Jinling Tang, Huiying Liang

**Affiliations:** 1 Institute of Pediatrics, Guangzhou Women and Children’s Medical Center, Guangzhou Medical University, Guangzhou, China; 2 Division of Epidemiology, The Jockey Club School of Public Health and Primary Care, The Chinese University of Hong Kong, New Territories, Hong Kong China

**Keywords:** Family dementia caregivers, Health and intervention, Global research trends, Bibliometric analysis

## Abstract

**Background:**

Dementia is a serious and growing health problem, and since most people with dementia live at home, caring responsibilities generally fall on family members. Caregivers are often inadequately supported by formal health services and have poorer psychological and physical health. Our study aimed to compare the contributions of publications from different countries, institutions and authors and present a bibliometric analysis to determine the hotspots and trends in research concerning the health of and interventions for family dementia caregivers.

**Methods:**

Studies published during 1988–2018 were extracted from the Science Citation Index Expanded of the Web of Science. Each abstract of publications was evaluated to obtain the basic information. A bibliometric analysis was used to evaluate the number or cooperation networks of publications, countries, institutions, journals, citations, authors, references, and keywords. The resulting articles were analyzed descriptively, and the publication keywords were visualized using VOSviewer.

**Results:**

Five hundred forty-two articles were identified. The annual number of relevant publications has steadily increased since approximately 2006. The USA has the highest number of publications (36.2%), followed by the UK (12.9%). China entered the field late, but research conducted in China has rapidly developed. The most productive institution, journal, and author in this field are University College London, the Journal of the American Geriatrics Society, and Orrell M from the UK, respectively. A co-occurrence analysis of the keywords reveals a mainstream research focus on burden, depression, quality of life, and corresponding interventions for people with dementia caregivers. The keywords “psychosocial intervention”, “long-term”, “e-learning/online”, “communication”, and “qualitative research” reflect the latest hotspots, appearing in approximately 2017–2018.

**Conclusion:**

Our study details the performance statistics, main topics and trends research on the health of and interventions for dementia caregivers from 1988 to 2018 and provides a comprehensive analysis.

## Introduction

The majority of persons with dementia living at home are cared for by their spouses, children, or friends, and family caregivers provide an estimated 80% of care [1, 2]. Due to the aging of the world population, the number of persons with dementia is expected to reach 75.6 by 2030 and 135.5 million by 2050 [3]; however, support services for caregivers are not expected to increase at the same rate [4].

The health of caregivers can be overlooked, and they have been referred to as ‘silent patients’ or ‘secondary patients’ [5, 6]. The negative outcomes associated with caregiving include depression and anxiety, and caregivers exhibit high rates of mood disorders [7]. The heavy burden and psychological distress caused by caring for a relative with dementia may also contribute to severe physical and psychiatric problems and higher mortality risk [8]. Recognizing this need, interventions have been developed to address the negative effects of caregiving, including education, respite, psychotherapeutic, family support, and multicomponent approaches [9]. In recent decades, the research on this topic has covered a wide spectrum. However, a detailed analysis of the existing research publication landscape has not been undertaken to elucidate the body of published evidence. Such an analysis would be helpful for delineating the global trends in the research fields related to the health of and interventions for dementia caregivers.

The aims of this study were to examine the publication patterns of research on the health of and interventions for family dementia caregivers at a global level. We attempted to provide comprehensive insights into the current state of global research on the health of and interventions for family dementia caregivers including annual output characteristics, international collaborations, and keyword trends. The results should provide a better understanding of the global trends in the research on the health of and interventions for family dementia caregivers and indicate directions for further research.

## Methods

The data were downloaded from public databases and, as secondary data, did not require any interactions with human subjects. There were no ethical issues associated with the data, and approval from institutional review boards was not required.

The data were obtained from the Science Citation Index-Expanded (SCI-E) of the Web of Science (WOS) on Jan. 6, 2019; publications published between 1988 and 2018 were retrieved. The keywords used in the search were as follows: (“dementia” OR “Alzheimer’s disease” OR “cognitive impairment”) AND (“carer” OR “caregiver” OR “spouse” OR “family care”) AND (“intervention” OR “support” OR “training” OR “programme” OR “program” OR “therapy” OR “education” OR “counselling” OR “respite” OR “psychosocial” OR “mentoring” OR “financial” OR “cognitive”). Two independent researchers (HS and HYL) conducted the search simultaneously to ensure that the search was accurate and that all relevant manuscripts were identified and included. The search results from the Web of Science database were subsequently filtered to include peer-reviewed articles and reviews in any language. These papers were published in English, French, German, Spanish and Italian, all of which could be read by our team using translation programs. The .txt data download from the WOS included the author names, contact address, title, publication year, abstract, author keywords, journal information, and cited references.

Bibliometrics is defined as the application of statistics and mathematics for the analysis of written publications such as books and journal articles [10]. Bibliometric techniques can be used to perform statistical and quantitative analysis of publications and offer a convenient way to visibly measure researchers’ efforts in the investigation of a specific field [11]. Such analyses can characterize the development in a certain field and have contributed substantially to informing policy making, clinical guidelines, and research trends [12]. Impact factors (IF) were taken from those published by the WOS in 2018. Cooperation among countries or institutions was based on the first author. Collaboration type was determined by the addresses of the authors, where the term “single-country article” was assigned if the researchers’ addresses were in the same country. The term “internationally collaborative article” was used to designate those articles that were coauthored by researchers from multiple countries [13, 14].

The data were analyzed both quantitatively and qualitatively. We describe the research status of this field based on statistics regarding the number of articles and their distribution across countries and in time and space, subjects, journals, and high-yield institutions and authors. Moreover, the corpus was analyzed using VOSviewer software to generate different scientific landscapes and networks. We analyzed the cooperation situation in this field among countries and institutions via cooperation analysis; the intrinsic links among authors via bibliographic coupling; and the reference network and frequently cited literature via co-citation analysis. In the hotspot analysis, we captured high-frequency keywords for the clustering and co-occurrence analyses. The bibliometric analysis in this study was performed and visualized using VOSviewer and bibliometric.com (http://bibliometric.com/).

## Results

### General information

**Fig. 1:**
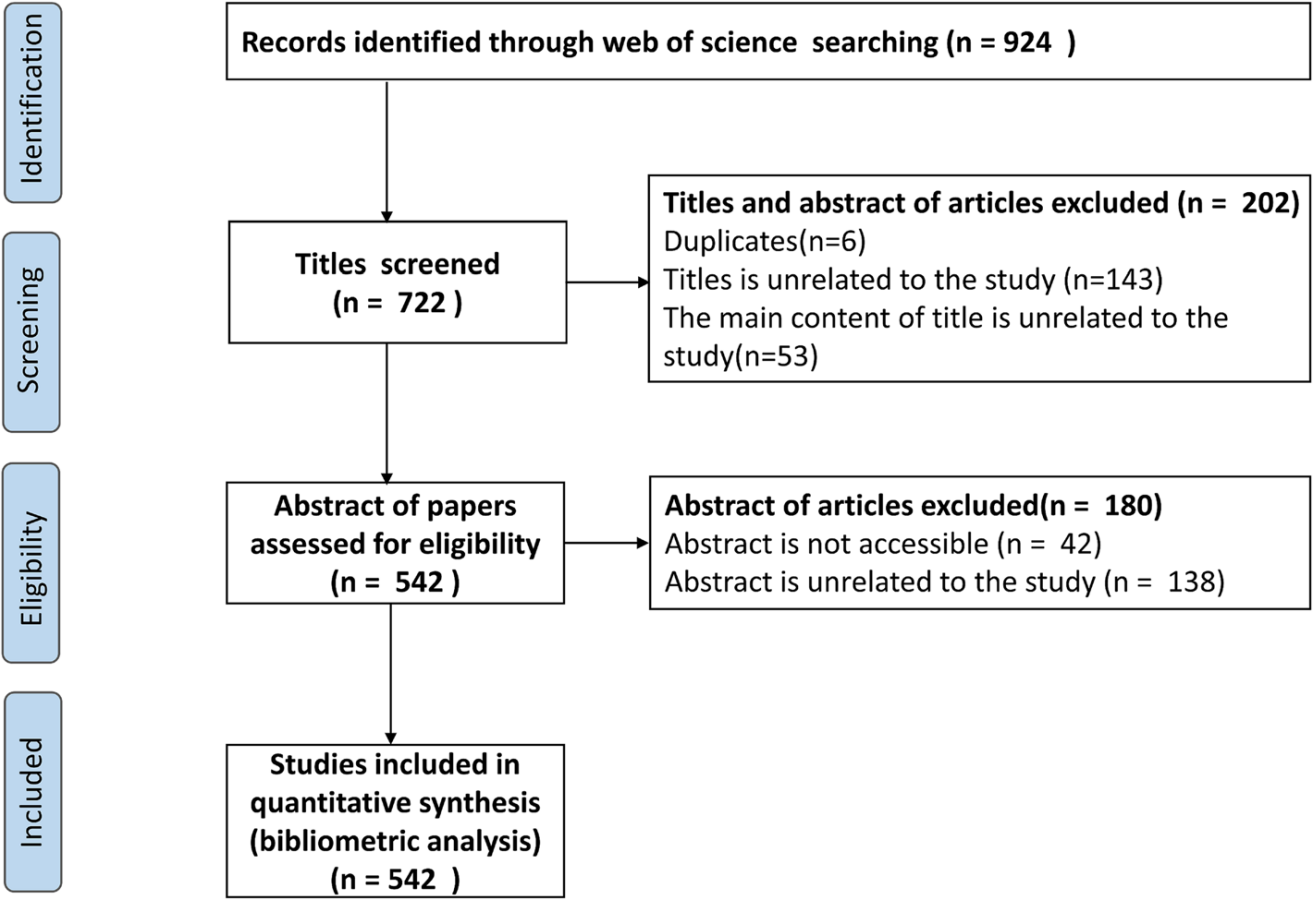
Flow diagram for the inclusion of publications on the health of and interventions for family dementia caregivers

**Fig. 2:**
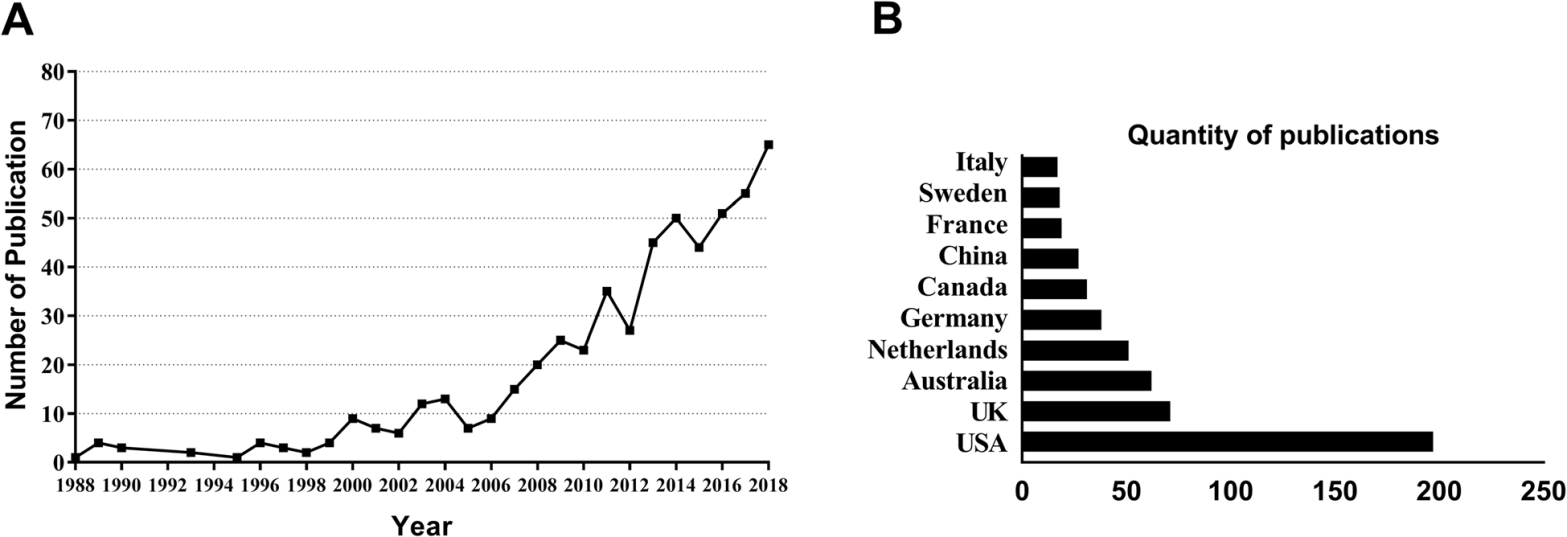
**a** The number of publications on the health of and interventions for family dementia caregivers by year, 1988–2018. **b** The number of publications on the health of and interventions for family dementia caregivers from the top 10 countries

In total, 924 publications on the health of and interventions for family dementia caregivers published between 1988 and 2018 were identified through our search strategy using the WOS. In total, 722 titles were excluded, and the final number of full-length articles was 542(Fig. 1). The papers were published by 2328 authors in 177 journals and used 2143 keywords (after de-duplication). The total number of citations was 11,289. The number of articles fluctuated during 1988–2000 and then began growing steadily in 2000. Of the included articles, 464 were published between 2006 and 2018, accounting for 85.6% (Fig. 2a).

### Country

The 542 articles were published in 43 countries, and the top 10 countries are shown in Fig. 2b. The USA is the clear leader in terms of publication quantity (196, 36.2%), followed by the UK (70, 12.9%), and Australia (61, 11.3%). Additional file 1: Figure S1A shows the changes in the number of publications over time and the national distribution characteristics, and Additional file 1: Figure S1B shows the international cooperation. The USA is not only a major producer in this research field but also has more collaborations, primarily with China.

### Institutions

The publications in this field were from 941 institutions. The top 10 institutions ranked by the number of articles published 141 articles, approximately 26.0% of the total (Table 1). The leading institutions that were most productive in terms of published articles were University College London, which published the greatest number (27, 5.0%), followed by Vrije University Amsterdam (19, 3.5%), and New York University (15, 2.8%). Among the top 10 institutions ranked by the number of articles published, four are located in the USA, two are located in the UK, two are located in the Netherlands, and another two are located in China and Australia. In addition, among the top 10 institutions with the highest total number of citation of publications, the University of New South Wales has the highest number of citations (783), followed by Columbia University (683) and Prince of Wales Hospital (673).

**Table 1:** The top 10 institutions ranked by the number and citation frequency of articles on the health of and interventions for family dementia caregivers published from 1988 to 2018

Rank	Institution	TP	TC	Rank	Institution	TP	TC
1	University College London,	27	464	1	University of New South Wales	8	783
2	Vrije University Amsterdam	19	276	2	Columbia University	8	683
3	New York University	15	581	3	Prince of Wales Hospital	6	673
4	King’s College London	14	492	4	New York University	15	581
5	Stanford University	14	347	5	King’s College London	14	492
6	University of Pittsburgh	13	348	6	University College London	27	464
7	University of Queensland	11	171	7	University of Alabama	6	457
8	Maastricht University	10	37	8	University of Manchester	8	381
9	Chinese University of Hong Kong	9	212	9	University of Toronto	7	359
10	Pennsylvania State University	9	317	10	Thomas Jefferson University	7	358

*TP* is the number of total publications, *TC* is the number of total citations

The top 30 institutions ranked by number of publications were subjected to a co-author analysis (Additional file 1: Figure S2). The network map shows which institutions have close cooperation, such as University College London and King’s College London; University College London and the University of Nottingham; the University of Pittsburgh and the University of Miami; New York University and Columbia University; Vrije University Amsterdam and the University of Queensland; and the Chinese University of Hong Kong and Hong Kong Polytechnic University.

### Journals

A total of 177 journals have published articles in this field. Nearly half of the publications were published in the top 10 journals (229, 43.7%) (Table 2). The *International Journal of Geriatric Psychiatry*, *International Psychogeriatrics*, and *Aging & Mental Health* published the most manuscripts. The *Journal of the American Geriatrics Society* shows the highest citation frequency per publication (64 times). The *International Journal of Geriatric Psychiatry* ranks second, with 27 citations per publication. This information, combined with the IF, indicates that the *Journal of the American Geriatrics Society* can be considered the most influential journal in the field of health intervention for dementia caregivers.

**Table 2:** The top 10 journals ranked by the number of articles on the health of and interventions for family dementia caregivers published from 1988 to 2018

Journal	IF	TP	TC	CPP
International Journal of Geriatric Psychiatry	3.14	42	1149	27.36
International Psychogeriatrics	2.48	42	560	13.33
Aging & Mental Health	2.95	36	629	17.47
Journal of The American Geriatrics Society	4.11	21	1338	63.71
American Journal of Geriatric Psychiatry	3.49	20	467	23.35
BMC Geriatrics	2.82	19	133	7.00
American Journal of Alzheimer’s Disease and Other Dementias	1.46	15	232	15.47
TRIALS	1.98	15	96	6.40
Clinical Interventions in Aging New Zealand	2.58	10	104	10.40
Dementia and Geriatric Cognitive Disorders	2.26	9	155	17.22

*IF* is the impact factor, *TP* is the number of total publications, *TC* is the number of total citations, *CPP* is the citations per publication

### Authors, articles and references

The top 10 authors contributed at first authors to a total of 44 papers related to health interventions for dementia caregivers, accounting for 8.1% of all relevant published studies (Table 3). Mittelman MS from New York University in the USA published the most papers as first author in this field (7 papers, cited 96 times), followed by Brodaty H from the University of New South Wales Drexel University in Australia (6 papers, cited 127 times), and Gaugler JE from the University of Minnesota (6 papers, cited 12times). All articles were subjected to a bibliographic coupling analysis (bibliographic couplings exceeded five) to produce the network map shown in Additional file 1: Figure S3. The connection between two nodes indicates that the articles have a common reference, and the weighting reflects the coupling strength of the two nodes. Thick connections indicate numerous common references between the two authors, that is, their study subjects are similar. For example, Pot AM, de Vugt ME, and Verhey FRJ, all highly productive authors with similar research directions.

**Table 3:** The top 10 authors ranked by the number of articles as first author on the health of and interventions for family dementia caregivers published from 1988 to 2018

Rank	Author	Affiliation	FP	FC
1	Mittelman MS	New York University	7	96
2	Brodaty, H	University of New South Wales	6	127
3	Gaugler, JE	University of Minnesota	6	12
4	Gitlin LN	Drexel University	5	21
5	Droes, RM	Vrije University Amsterdam	4	12
6	Kuo, LM	National Taipei University	4	2
7	Graff, MJL	Radboud University	3	32
8	Garand, L	University College London	3	27
9	Livingston, G	University College London	3	23
10	Nichols, LO	University of Tennessee	3	22

*FP* is the number of total publications as first author, *FC* is the number of total citations as first author

The ten most-cited articles in this field are presented in Additional file 1: Table S1. The paper entitled “Meta-analysis of psychosocial interventions for caregivers of people with dementia” (2010), published in the *Journal of the American Geriatrics Society* by Brodaty H et al., was the most frequently cited, with 497 citations.

Twenty-four references with a citation frequency greater than 30 were selected to create the co-occurrence map, as shown in Additional file 1: Figure S4, where each node represents a highly cited article. A large node indicates that the article has a high citation frequency. The connection between two nodes indicates co-citation, the thickness of the connection line reflects the intensity, and the Euclidean distance of the two nodes indicates the articles’ similarity. The reference titled “A Practical Method for Grading the Cognitive State of Patients for the Clinician”, published by Folstein MF in 1973, was the most frequently cited article.

### Hotspots

**Fig. 3:**
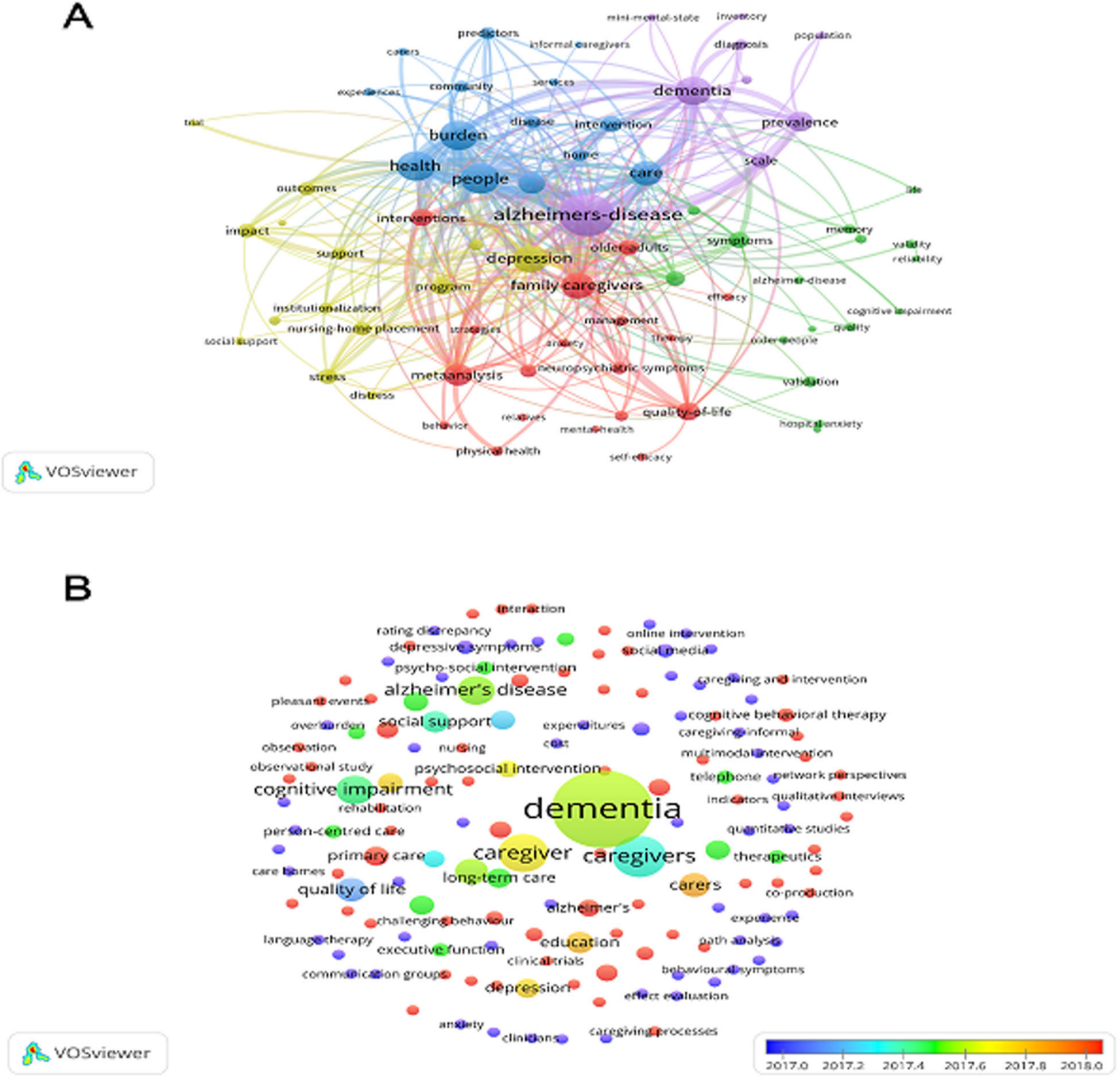
**a** Mapping of the keywords; the keywords were divided into five clusters shown in different colors generated by default: burden-related research (top left corner in blue), depression- and stress-related research (left in yellow), quality-of-life-related research (bottom center in red), methods and symptoms (right in green) and description and prevalence (top right corner in purple). **b** Distribution of keywords according to the average time they appeared in the literature in 2017–2018. Key words in blue were presented earlier than those in red

The keywords of the 542 papers were analyzed using VOSviewer. As shown in Fig. 3a, the 71 keywords (those used more than 10 times in the abstracts of all articles) were classified into the following five clusters: “Burden-related research” (top left corner in blue), “Depression- and stress-related research” (left in yellow), “Quality-of-life-related research” (bottom center in red), “Methods and symptoms” (right in green) and “Description and prevalence” (top right corner in purple).

Additional file 1: Table S2 shows the 71 keywords. In the “Burden-related research” cluster, the most commonly used keywords were “burden” (104 times), “health” (105 times), and “intervention” (46 times). In the cluster “Depression- and stress-related research”, the most commonly used keywords were “depression” (112 times), “stress” (36 times), and “impact” (35 times). In the cluster “Quality-of-life-related research”, the most commonly used keywords were “meta-analysis” (65 times), “quality of life” (48 times), and “psychosocial interventions” (29 times). In the cluster “Methods and symptoms”, the most commonly used keywords were “symptoms” (42 times), “randomized controlled trial” (37 times), and “validation” (23 times). In the cluster “Description and prevalence”, the most commonly used keywords were “Alzheimer’s disease/dementia” (282 times), “prevalence” (56 times), and “scale” (38 times). Publication with the keywords “burden”, “depression”, “stress”, “quality of life” focused on health of family caregivers, while publication with the keywords “psychosocial interventions”, “intervention” focused on interventions for the target group. Furthermore, the map of the average keyword frequency by year (Additional file 1: Figure S5) shows that “depression” and “burden” were the focus of early studies and have been the most widely studied keywords, and research related to these topics begin in approximately the year 2000. Outcomes such as “self-efficacy”, “behavioral problems” and “quality of life”, gradually emerged in the following years, indicating that researchers began to pay attention to broader physiological and mental health problems.

We further analyzed the research hotspots in this field in 2017 and 2018, and VOSviewer applied colors to the keywords based on the year (2017 or 2018) that they appeared in the literature (Fig. 3b). The results show that the keywords “psychosocial intervention” (6 times), “long-term” (5 times), “e-learning/online” (5 times), “communication” (5 times), “qualitative research” (5 times), “cognitive behavioral therapy” (4 times), “interaction/mixed methods” (3 times), and “music therapy” (3 times) may indicate the main research hotspots in recent years.

## Discussion

Demographic ageing is a worldwide process and is associated with an increased prevalence of Alzheimer’s disease [1]. It is inevitable that family caregivers and caregiver-related problems will increase, because most people with dementia live at home [6]. When the 10/66 ‘Helping Carers to Care’ Research Group (funded by Alzheimer’s Disease International) was founded in 1998, the study of informal caregivers for dementia became more extensive. Our results support this trend, i.e., research concerning health interventions for dementia caregivers started to increase in approximately 2000 (Fig. 2a). The World Alzheimer Report 2013 states that the number of people with dementia in need of care is rapidly increasing in developing countries [15], however, in our study, most articles are form developed countries, mainly the USA, the UK and Australia. They dominate this field of research, as of the top 10 most active institutions, 3 are in the US and 2 in the UK. National policies and support for dementia may play an important role in this phenomenon. For example, in 2004, Australia was the first country to declare dementia a national health priority, and national dementia strategies have been launched in France, South Korea, England, Norway and the Netherlands [7]. It is noteworthy that China is the only developing country among the top 10 most productive countries and that the Chinese University of Hong Kong is also a high-yield institution. China and the United States closely cooperate and have conducted numerous studies in different regions encompassing the racial, cultural, economic and social diversity of the nation overall [16], indicating that China has begun to make important contributions to this field.

Further analysis shows that the most closely cooperating institutions are located in the same country and that cooperation among countries is not very common. Furthermore, compared to other fields, such as oncology, not enough research focuses on family caregivers of people with dementia, and high-quality evidence is limited. The variation in health care systems, culture, family structure and care arrangements across countries and regions can lead to different health problems and countermeasures for caregivers, which may be reasons for the lack of international cooperation in this type of research. Despite the call by the WHO for an international discussion regarding how to provide caregiver protection [15].

The *Journal of the American Geriatrics Society* can be considered the most influential journal in this field, and it focuses on geriatrics and gerontology. We observed that no articles were published in the top-tier general medical journals, and of the 10 most-cited articles, four articles were randomized controlled trials. Nearly half of the publications related to the health of and intervention for family caregiver of people with dementia were randomized controlled trials, but the review found mixed methodological quality and mixed results in terms of some intervention outcomes [17, 18]. Incomplete data reporting, high dropout rates, and recruitment are remaining challenges in study designs in this field [19].

The hotspot analysis results show that burden, depression/stress and quality of life are the most widely studied issues. Studies have shown that approximately 30–55% of caregivers suffer from anxiety or depressive symptoms [20, 21], which may result in changes in their behavior and reduced quality of care. Although the existing outcome measures predominately focus on the negative aspects of caregiving, the positive aspects of caring, such as self-efficacy, resilience, rewards, meaning and spirituality, have been overlooked [22]. The positive aspects of caring could aid in the development of relevant positive psychology interventions [23]. Based on the above caregiver health issues, several international agreements and conventions were drafted by the WHO and Alzheimer’s Disease International (ADI), and some countries also have policies protecting the rights of caregivers, such as the 10/66 ‘Helping Carers to Care’ train-the-trainer intervention in ADI [24], a national strategy for carers and Care Act in the UK [25–28], and the national dementia strategies in Australia. However, most countries/ regions still do not have long-term care (LTC) services and support for family caregivers of people with dementia, such as China [29]. Furthermore, research institutes in various countries have conducted extensive research investigating interventions for dementia caregivers’ health issues. Silvia Sörensen and colleagues catrgorized the following six types of caregiver intervention: pychoeducational interventions, supportive interventions, respite/adult day care, psychotherapy, interventions to improve care receiver competence, and multicomponent interventions [30]; our results also show “social support”15 times and “psychosocial interventions” 29 times in 542 articles. Additionally, our study show that studies are increasingly conducted via the “telephone” and “Internet” (beginning in approximately 2014). The keywords “psychosocial intervention”, “e-learning/online”, “interaction/mixed methods”, “music therapy”, etc. began to appear in 2017 and 2018. The use of Internet-based interventions seems to have great potential [31] and represents a trend for future research.

There were some limitations in this study. First, the data were extracted only from the WOS database. Second, there were differences between the real research conditions and the bibliometric analysis results, since some recently published studies were not included. Furthermore, the bibliometric mapping (term association analysis) and thematic analysis were performed on the abstracts of the information sources only; it is thus possible that the results could have been different if the publications had been analyzed in their entirety (if the complete articles were available).

## Conclusion

An overview of research on health interventions for dementia caregivers was presented, providing information related to the number of annual publications, countries, journals, institutions, authors and research trends. There is an urgent global unmet need for effective scalable interventions for caregivers of people with dementia, given the fast moving pace of this research area. We believe that the key to improve health of family caregivers of people with dementia lies in a unique combination of global solutions and local knowledge. International cooperation should be strengthened to solve this problem. Future research needs the generalisation of treatment effects in different countries and carers of patients with different types of dementia need to be addressed. Furthermore, it is evident that RCT evidence is only a part of the current body of evidence; more attention to non-RCT evidence may substantially help future trial designs, and a realist review could be undertaken to enhance our understanding of the complexity of the implementation and impacts of interventions.

In summary, we identified and analyzed the characteristics of the research on the health of and interventions for family dementia caregivers. The results may help guide researchers and funding agencies to the most important research areas in the field.

## Supplementary information

**Additional file 1: Table S1.** The top 10 articles ranked by the citation frequency of articles on the health of and interventions for family dementia caregivers published from 1988 to 2018. **Table S2.** The 71 keywords in five domains. **Figure S1.** (A) The top 10 countries’publications on the health of and interventions for family dementia caregiversby year, 1988–2018 (B) Interactions between countries of the included publications. **Figure S2.** Cooperation among the top 30 institutions, 1988–2018. **Figure S3.** Bibliographic coupling by author, 1988–2018. **Figure S4.** Co-occurrence map of the most-cited articles. **Figure S5.** Distribution of keywords according to the average time they appeared in the literature. Key words in blue were presented earlier than those in yellow.

## Data Availability

All data generated or analyzed during this study are included in this published article and its supplementary information files.
