# Supplementary material for: Research on the health of and interventions for family caregivers of people with dementia: a bibliometric analysis of research output during 1988–2018
Source: BMC Geriatr. 2020 Jan 21;20:20. doi: 10.1186/s12877-020-1421-7 (PMC6975077; doi:10.1186/s12877-020-1421-7)
Supplement: Supplementary file 1 — Additional file 1: Table S1. The top 10 articles ranked by the citation frequency of articles on the health of and interventions for family dementia caregivers published from 1988 to 2018. Table S2. The 71 keywords in five domains. Figure S1. (A) The top 10 countries’publications on the health of and interventions for family dementia caregiversby year, 1988–2018 (B) Interactions between countries of the included publications. Figure S2. Cooperation among the top 30 institutions, 1988–2018. Figure S3. Bibliographic coupling by author, 1988–2018. Figure S4. Co-occurrence map of the most-cited articles. Figure S5. Distribution of keywords according to the average time they appeared in the literature. Key words in blue were presented earlier than those in yellow. [file 12877_2020_1421_MOESM1_ESM.pdf]

Supplementary

Table S1. The top 10 articles ranked by the citation frequency of articles on the health of and interventions for family dementia caregivers published from 1988 to 2018.

Table S2. The 71 keywords in five domains.

Figure S1. (A) The top 10 countries' publications on the health of and interventions for family dementia caregivers by year, 1988-2018 (B) Interactions between countries of the included publications.

Figure S2. Cooperation among the top 30 institutions, 1988-2018.

Figure S3. Bibliographic coupling by author, 1988-2018.

Figure S4. Co-occurrence map of the most-cited articles.

Figure S5. Distribution of keywords according to the average time they appeared in the literature. Key words in blue were presented earlier than those in yellow.

Table S1. The top 10 article ranked by the citation on health and intervention for family dementia caregivers from 1988 to 2018.

Rank	Article details	Citations
1	**Brodaty H, Green A, Koschera A**. Meta-analysis of psychosocial interventions for caregivers of people with dementia. [J]. Journal of the American Geriatrics Society, 2010, 51(5):657-664.	497
2	**Graff M J L, Vernooijdassen M J M, Thijssen M, et al.** Community based occupational therapy for patients with dementia and their care givers: randomised controlled trial **[J]. Bmj, 2006, 333(7580):1196-1199.**	228
3	**Mittelman M S , Roth D L , Coon D W , et al**. Sustained Benefit of Supportive Intervention for Depressive Symptoms in Caregivers of Patients With Alzheimer's Disease[J]. American Journal of Psychiatry, 2004, 161(5):850-856.	194
4	**Brodaty H, Gresham M**. Effect of a training-program to reduce stress in carers of patients with dementia [J]. BMJ Clinical Research, 1990, 299(6712):1375-1379.	189
5	**Thommessen B, Aarsland D, Braekhus A, et al.** The psychosocial burden on spouses of the elderly with stroke, dementia and Parkinson\"s disease **[J]. International Journal of Geriatric Psychiatry, 2002, 17(1):78-84.**	143
6	**Gitlin L N, Winter L, Dennis M P, et al.** A Biobehavioral Home-Based Intervention and the Well-being of Patients With Dementia and Their Caregivers: The COPE Randomized Trial **[J]. The Journal of the American Medical Association, 304.**76-f6276.	139
7	Hepburn K W, Tornatore J, Center B, et al. Dementia family caregiver training: Affecting beliefs about caregiving and caregiver outcomes [J]. Journal of the American Geriatrics Society, 2001, 49(4):450-457.	137
8	**Mittelman M S , Roth D L , Haley W E , et al.** Effects of a Caregiver Intervention on Negative Caregiver Appraisals of Behavior Problems in Patients With Alzheimer's Disease: Results of a Randomized Trial [J]. The Journals of Gerontology Series B: Psychological Sciences and Social Sciences, 2004, 59(1):P27-P34.	135
9	**Brodaty H, Hadzi-Pavlovic D**. Psychosocial Effects on Carers of Living with Persons with Dementia [J]. Australian and New Zealand Journal of Psychiatry, 1990, 24(3):351-361.	132
10	**Graff M J L , Vernooij-Dassen M J M , Thijssen M , et al**. Effects of Community Occupational Therapy on Quality of Life, Mood, and Health Status in Dementia Patients and Their Caregivers: A Randomized Controlled Trial[J]. The Journals of Gerontology Series A: Biological Sciences and Medical Sciences, 2007, 62(9):1002-1009.	119

Table S2. The 71 keywords in five domains.

Domains	keywords
"Burden-related research"	people(109), health(105), Burden(104), care(80), intervention(41), disease(30), predictors(27), community(24), services(14), carers(13), experiences(15), informal caregivers(11).
"Depression-related research"	depression/depressive symptoms (123), program(44), stress(36), impact(35), nursing-home placement(31), outcomes(30), support(27), institutionalization(22), distress(18), spouse-caregivers(18), social support(15), trial(10).
"Quality-of-life-related research"	family caregivers/ caregivers (143), meta-analysis(65), quality-of-life(59) ,old-adults(51), interventions(46), psychosocial interventions(29), symptoms(28), management(20), strategies(17), physical health(19), neuropsychiatric behavior(16), anxiety(15), relatives(14), self-efficacy (13), mental-health(12), satisfaction(10).
"Method and symptoms"	symptoms(42), validation(23), memory(19), validity(17), quality(17), Alzheimer-disease(14),spital anxiety(14), older-people(12), life(11) reliability(11), cognitive impairment(10).
"Description and prevalence"	Alzheimer-disease(199), dementia(97), prevalence(56), randomized controlled-trial/ controlled-trial (56), older-adults(51), scale(38), home(27), experience(25), diagnosis(21), individuals(23), mini-risk(17), patient(16), efficacy(13), population(13), mental-state(12), therapy(12), inventory(11).

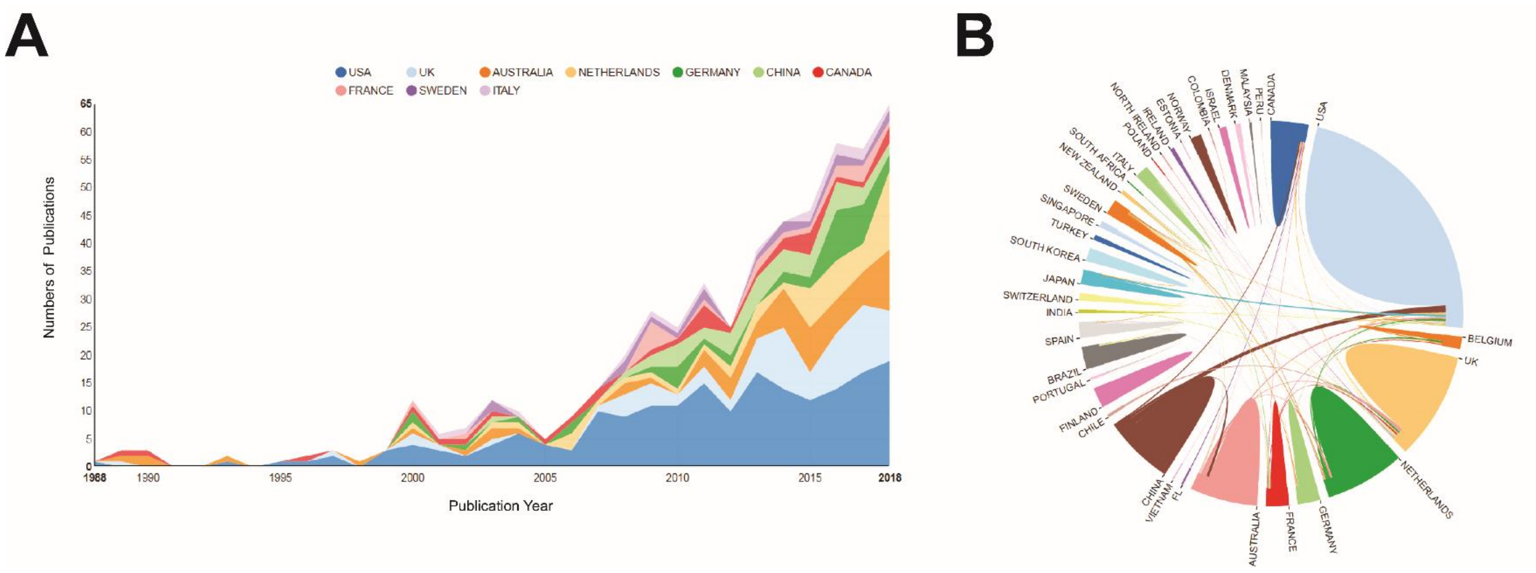

Figure S1. (A) The top 10 countries' publications on the health of and interventions for family dementia caregivers by year, 1988-2018 (B) Interactions between countries of the included publications.

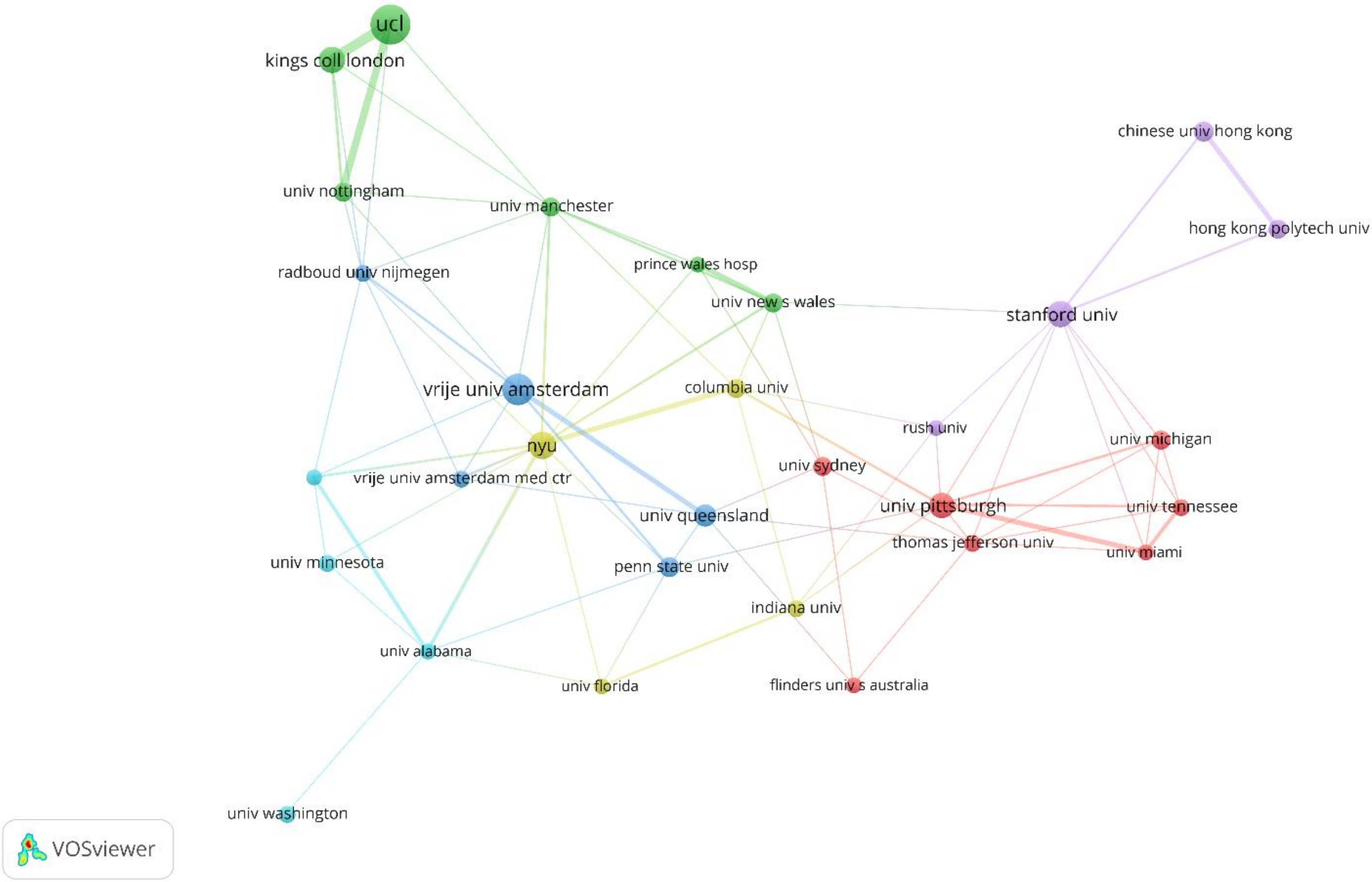

Figure S2. Cooperation among the top 30 institutions, 1988-2018.

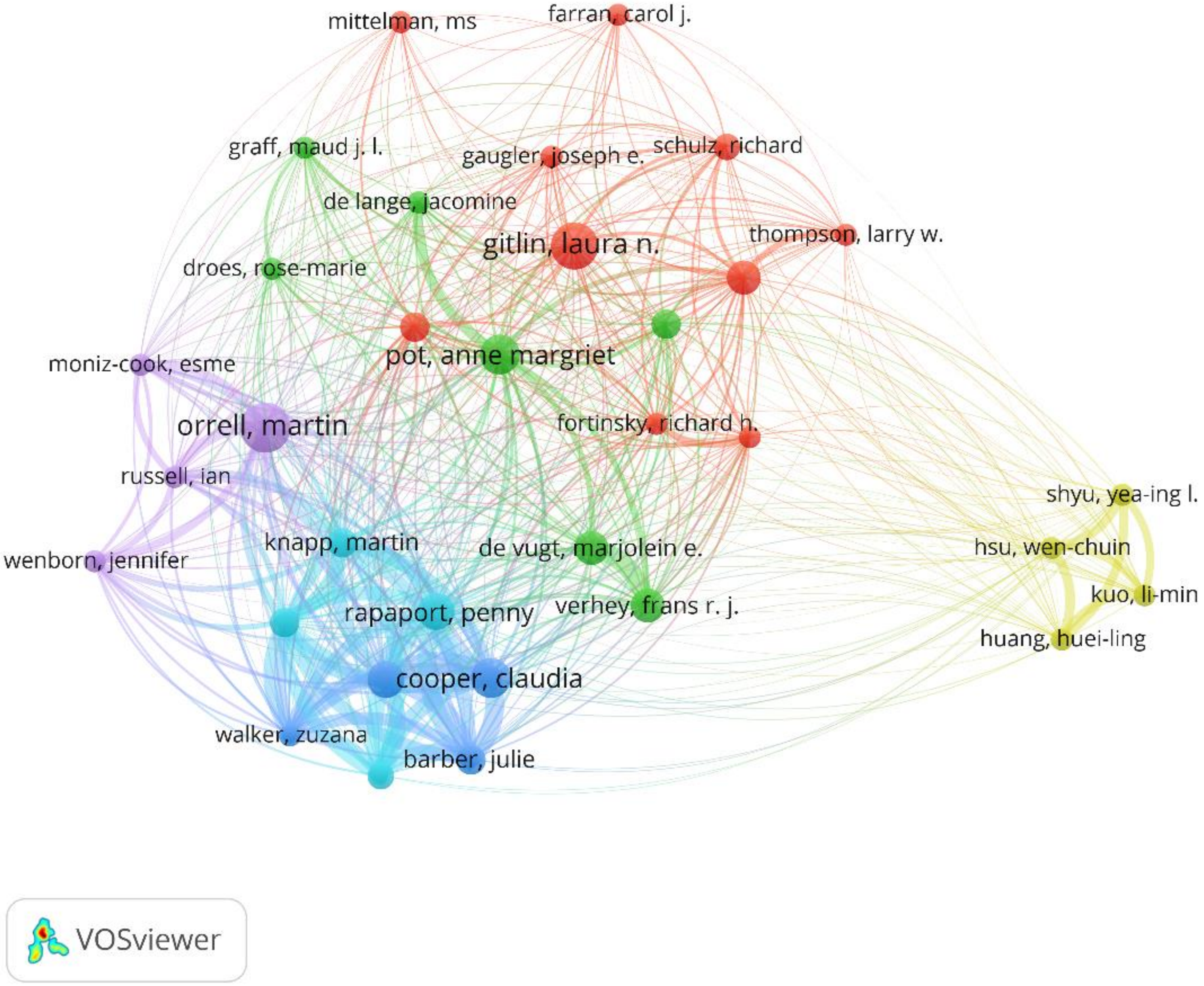

Figure S3. Bibliographic coupling by author, 1988-2018.

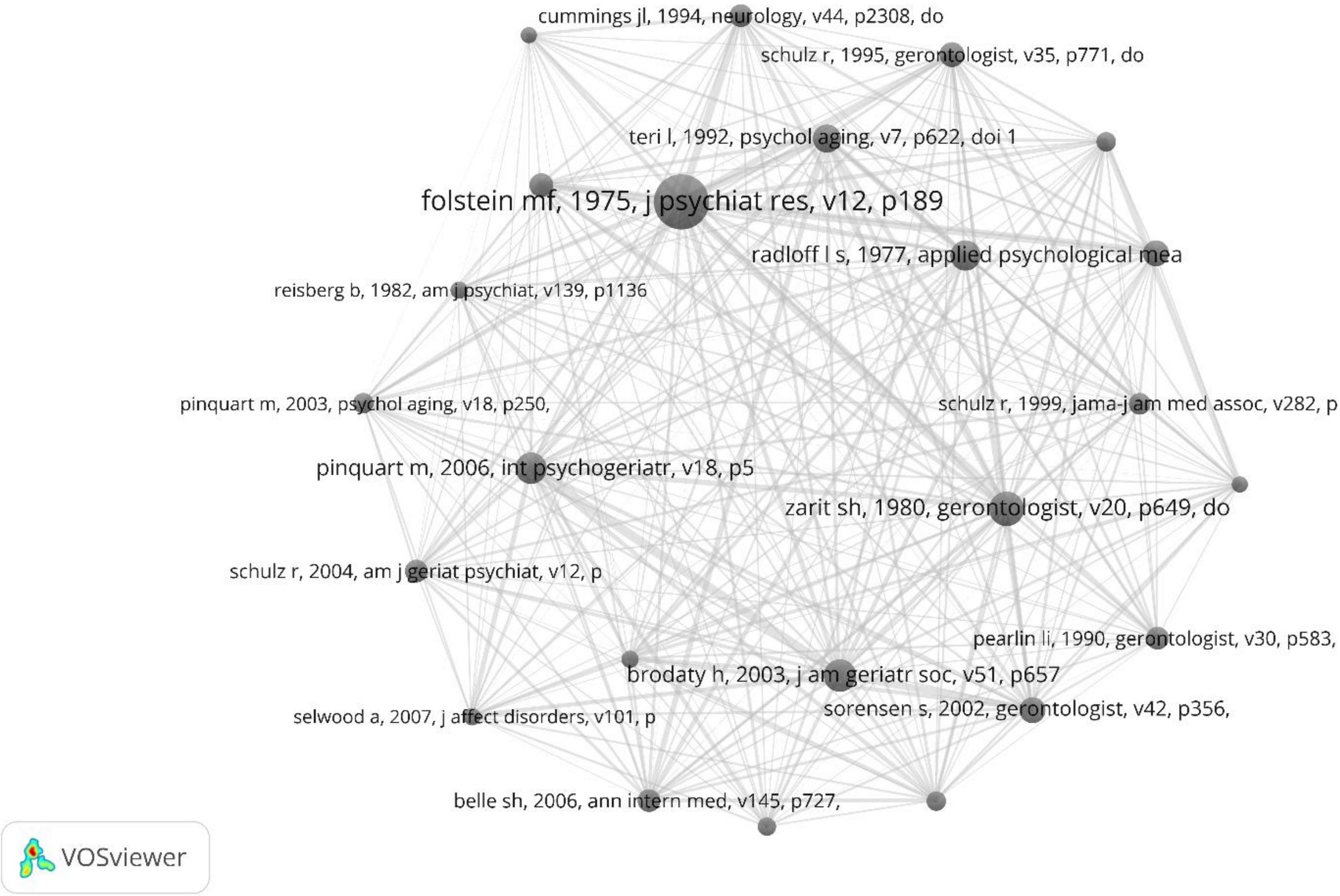

Figure S4. Co-occurrence map of the most-cited articles.

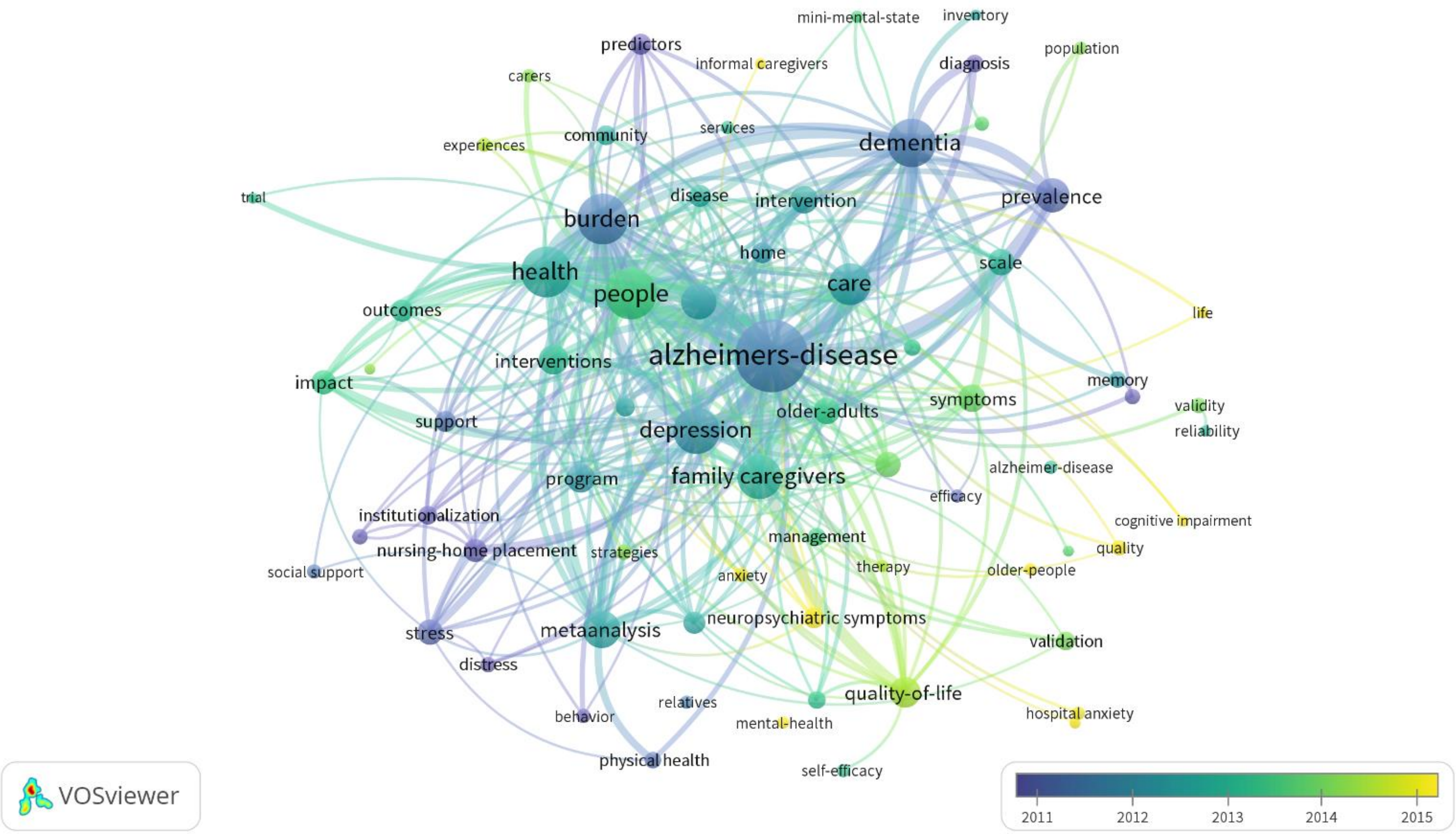

Figure S5. Distribution of keywords according to the average time they appeared in the literature. Key words in blue were presented earlier than those in yellow.
